# Supporting ALL victims of violence, abuse, neglect or exploitation: guidance for health providers

**DOI:** 10.1186/s12914-018-0178-y

**Published:** 2018-10-19

**Authors:** Roderik F Viergever, Nicki Thorogood, Judith RLM Wolf, Mary Alison Durand

**Affiliations:** 1 Department of Health Services Research and Policy, London School of Hygiene and Tropical Medicine, Tavistock Place, London, WC1H 9SH UK; 2 CoMensha, Amersfoort, the Netherlands; 3 Department of Public Health, Environments and Society, London School of Hygiene and Tropical Medicine, Tavistock Place, London, WC1H 9SH UK; 4 Impuls, the Netherlands Center for Social Care Research, Radboud university medical center, Nijmegen, the Netherlands

**Keywords:** Vulnerability, Vulnerable groups, Victims of violence, Human trafficking, Homeless people, Policy, Honour based violence, Violence, Female genital mutilation, Abuse, Parent abuse, Neglect, Exploitation, Slavery, Elder abuse, Forced marriages, Abuse by carers, Male victims, Grooming

## Abstract

Smaller groups of victims of violence, abuse, neglect or exploitation – such as male victims of intimate partner violence (IPV), victims of elder abuse, victims of abuse by carers, victims of parent abuse, victims of human trafficking, girls and boys below 18 years engaging in sex work, victims of sexual exploitation by gangs or groups and victims of honour based violence (such as forced marriages and female genital mutilation) – are often in contact with the health care system without being identified as such and frequently do not receive appropriate treatment. To address this problem, two things need to happen: 1) that ALL groups of victims of violence, abuse, neglect or exploitation are explicitly listed in policies and protocols, and 2) that both the similarities as well as the differences between the groups with regard to identification, support and referral – described in this article – are explained, so that health providers are appropriately supported in this important function.

**Electronic supplementary material:**

The online version of this article (10.1186/s12914-018-0178-y) contains supplementary material, which is available to authorized users.

## Background: Lesser-known victims of violence, abuse, neglect or exploitation

A positive development in global public health and social policy in the past few decades has been the recognition of the importance of adequate responses to victims of violence, abuse, neglect or exploitation (‘VANE’, from hereon). This has led to much improved policies and practices supporting female victims of intimate partner violence (IPV) and victims of child abuse. However, other specific groups of victims of VANE are often neglected in current policies and service delivery. As a result, health providers lack knowledge of these lesser-known groups, often meaning victims are in contact with the health system without being identified as such [1, 2] and frequently do not receive appropriate treatment [1–3]. E.g., while up to 88% of victims of human trafficking encounter a health provider while being trafficked, less than 20% of providers knows enough to identify or appropriately support them [4, 5]. Further examples of these often-neglected groups are provided in Table 1, in addition to IPV against women and child abuse. The table also lists their global and UK one-year prevalence.

**Table 1:** One-year prevalence data of different types of violence, abuse, neglect or exploitation (VANE) worldwide and in the UK

Type of violence, abuse, neglect or exploitation (VANE)	Available one-year prevalence data		
	*Worldwide (estimates)*	*in the UK (estimates)*	*in the UK (reported cases)*
*Specific groups of victims*			
Intimate partner violence (IPV)	1.4 billion (women only)	2.0 million	102,970^a^
IPV against men	–	716,000	40,985^a^
Child abuse	1.1 billion	520,000^b^	58,239
Elder abuse	141.4 million	342,400	65,085^a^
Abuse by carers	–	–	23,428^c^
Parent abuse	–	–	3339
*Specific type of VANE: Human trafficking and sexual exploitation*			
Human trafficking / forced labour	24.9 million	10–13,000	3805
Girls and boys below 18 years engaging in sex work	–	11,570^d^	–
Human trafficking: domestic, within-country	19.2 million	5618–7303^d^	326
Sexual abuse or exploitation by gangs or groups	–	–	2067^e^
*Specific type of VANE: Honour based violence*			
Honour based violence	–	–	2349
Forced marriages	15.4 million	–	1428
Female genital mutilation	3 million	154–193^d,f^	18

Other types of VANE that were considered for this table but were not included for various reasons are: violence against unborn children; children who witness domestic violence; children whose parents are in a violent divorce; stalking; boundary-crossing sexual behaviour among youths/children; IPV against/among vulnerable migrants (e.g., undocumented people, refugees and asylum seekers); online sexual intimidation (e.g., shame-sexting, grooming, revenge porn, sextortion, spreading images of sexual violence online, and sending or posting unsolicited messages of a sexual nature); financial exploitation; sexual violence; bullying; self-harm; and people at risk of radicalisation. ‘Estimates’ are estimates of ‘real’ one-year prevalence made on the basis of scientific models and/or experience. ‘Numbers of recorded cases’ are numbers of recorded, reported or confirmed cases over one year and are likely a strong underestimation. Data are from various years. Some groups may overlap partially with others. “-” means no data were found. For additional information about the numbers and sources, see additional file 2

^a^Number is for England only

^b^Numbers for “child maltreatment” are reported here, in line with definitions used by the UK National Society for the Prevention of Cruelty to Children (NSPCC). The number only pertains to maltreatment by a parent or guardian

^c^This number concerns abuse by contracted home carers, however, definitions of this abuse vary: it is defined by others as being about abuse by informal caregivers. Numbers for abuse by informal caregivers were not found. Abuse by carers differs from elder abuse in that it is not limited to abuse of elders, but may involve anyone who is cared for

^d^No data found for the UK; estimate based on estimated number of cases in the Netherlands (adjusted for population size)

^e^This number is based on confirmed cases from 20 out of 39 police constabulary areas in the UK; 19 areas did not provide data. Also: this number only concerns girls and boys below 18 years of age

^f^This is an estimate for the one-year prevalence of girls at risk of female genital mutilation (FGM). Estimates for the total number of women affected by FGM (137,000) and recorded requests for help regarding FGM (1564 over 3.5 years) are much higher, since these numbers include cases of FGM that took place in the past

## Main text: Similarities and differences between various groups of victims of VANE

**Fig. 1:**
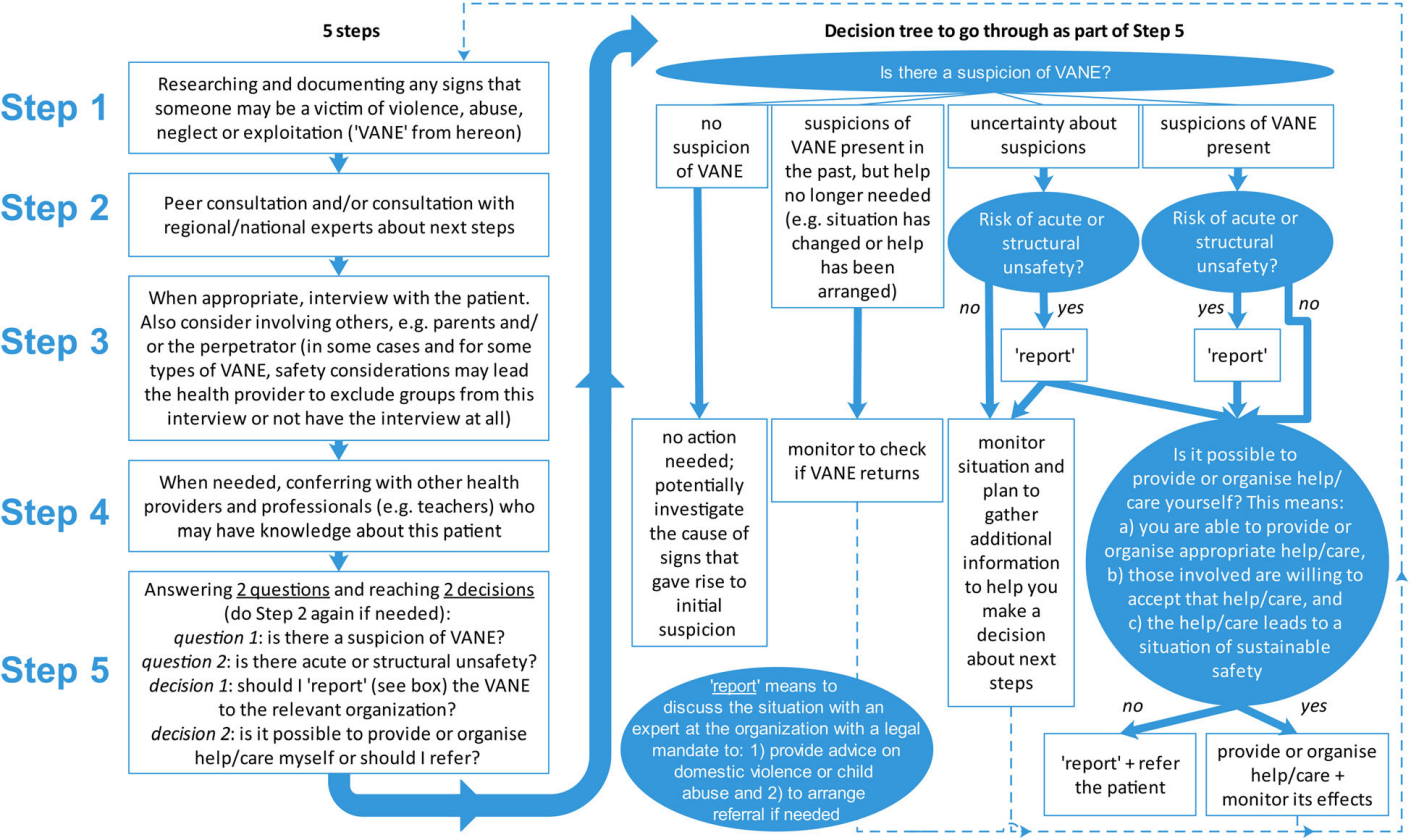
Five steps for deciding how to respond as a health provider when you suspect someone is a victim of violence, abuse, neglect or exploitation (VANE). This figure is based on the identification, support and referral protocol in the Netherlands; [6–8] other countries may have their own protocols that may differ from the one described here. In the Netherlands, the organization with a legal mandate to 1) provide advice on domestic violence or child abuse and 2) arrange referral when needed, is “Veilig Thuis”, meaning “Safe at Home”. Whenever possible, ‘reporting’ should be done with the consent of the patient, but when the health provider is of the opinion there is acute and/or structural unsafety, he/she may be justified or even obligated to break confidentiality and report the situation to this organization against the wishes of the patient

It is key that health providers recognize both the similarities and differences between these groups. Similar steps may be taken to a) identify, b) support and c) refer victims (if needed) (Fig. 1):

1. researching and documenting signs of VANE (including asking about VANE in the first place – that’s ‘step 0’ in this model);
2. consulting peers and experts;
3. deciding if it is appropriate to interview the patient (and/or others involved) and if yes, doing so;
4. conferring with other professionals who know the patient (e.g., teachers); and
5. reaching decisions about whether:
  A) the situation needs to be ‘reported’, meaning that the health provider chooses to discuss the situation with an organization with a legal mandate to 1) provide advice on domestic violence or child abuse and 2) arrange referral when needed (‘reporting’ is mandatory in some countries for certain types of cases of VANE. E.g., in the Netherlands, this is mandatory when i) there is (risk of) acute or structural unsafety, or ii) when the health provider is not able to provide or organise help/care him−/herself. This ensures that VANE professionals are involved in decision-making processes when needed);
  B) care/help can be provided by the health provider him−/herself or whether the patient needs to be referred.
    To be able to make these two decisions, two questions need to be answered about the situation: 1) Is there a suspicion of VANE? and 2) is there acute or structural unsafety? [6–8].

Recognizing that these general steps are the same for all types of VANE makes it easier for providers to respond appropriately to lesser-known types, as they can follow the same protocol.

At the same time, there are differences between the various groups of victims that providers need to take note of (Additional file 1). In terms of identification, the signs of VANE that could lead to identification differ by group, e.g., the signs of honour-based violence are quite different to those for IPV. Similarly, the risk factors that make someone more likely to be a victim differ by group, e.g., specific risk groups are known for female genital mutilation and parent abuse. Besides this, there are specific identification, support and referral considerations for most groups, around, for example:

- assessing safety, e.g., with human trafficking criminal networks may be involved leading to different safety assessments;
- breaking confidentiality, e.g., considerations around breaking confidentiality to ‘report’ VANE are different for VANE against adults than for child abuse [7];
- urgency, e.g., victims of some types of VANE, such as parent abuse and honour-based violence, typically present very late, necessitating swift action;
- communication, e.g., with some types of VANE, such as honour-based violence or cross-border human trafficking, if patients do not speak your language, it is important to speak to them via an independent translator when they are accompanied by someone (it should be the policy to speak to a potential victim alone for all types of VANE).

In many countries, protocols are available, either at national or local level, to guide health providers and social workers in a) identifying, b) supporting and c) referring victims (if needed). Unfortunately, these protocols are often limited in the types of VANE that they cover [7]. Explicitly including ALL types of VANE in such protocols increases the awareness of lesser-known groups, makes it easier for providers to respond appropriately to all types of victims using one protocol (Fig. 1) and makes providers take note of the differences between the groups of victims (Additional File 1). Therefore, at a policy level, explicitly including all groups of victims of VANE in relevant policies and protocols is key in improving standards of care for ALL the groups in Table 1.

This may be aided by using a more inclusive umbrella term to describe these groups. Currently, umbrella terms that are used frequently are ‘domestic violence’ (this is limited to violence by family members or other persons in a domestic setting) and ‘IPV’ (this is limited to violence between partners), excluding victims of violence by people outside the home (e.g., abuse by gangs or VANE by someone in the workplace) or by non-partners respectively. Also, the term ‘violence’ is often interpreted quite narrowly, e.g., as meaning only physical violence (although arguably a more broad interpretation is possible); therefore, it is better to speak about ‘violence, abuse, neglect or exploitation’. These exclusions are missed opportunities, because clearly it is beneficial for victims of *any* type of VANE if health providers (or other professionals) are able to a) identify, b) support and, if needed, c) refer them appropriately. To address some of these problems, the Netherlands have opted to use the term ‘Violence in power-imbalanced relationships’, [9] which includes all types of VANE in any relationship (parents, partners, teachers, health providers, work colleagues, etcetera), regardless of setting – that is, all groups in Table 1. By using the word ‘power-imbalanced’ this term stresses that victims of VANE often have an unequal power relationship with the perpetrator. Although the word ‘violence’ in this term is mostly broadly interpreted in the Netherlands as meaning VANE, a further improvement may be to speak of ‘*Violence, abuse, neglect or exploitation in power-imbalanced relationships*’.

## Conclusion

Sustainable Development Goals (SDGs) 5 and 16 ask that we aim to end ALL forms of violence, neglect, abuse and exploitation. To this end, we recommend health ministries and professional health provider organizations ensure two things happen: 1) that all groups of victims of VANE are explicitly listed in policies and protocols, and 2) that both the similarities (e.g., Fig. 1) as well as the differences (e.g., Additional file 1) between the groups with regard to identification, support and referral are explained, so that health providers are appropriately supported in this important function. It may also be beneficial to adopt a more inclusive umbrella term to describe all types of VANE together.

### Notes

We would like to note that in speaking about ‘health providers’ in this article we mean the broadest possible range of health and social care workers, including, for example, all medical and nursing professions, physiotherapists, dentists, midwives and social workers.

## Additional files

Additional file 1: Examples of differences between smaller groups of victims of violence, abuse, neglect or exploitation (VANE) in terms of identification, support and referral: an overview for health providers and social workers. (DOCX 36 kb)

Additional file 2: Sources used in the development of Table 1. (DOCX 47 kb)

## Abbreviations

IPV: Intimate partner violence
SDGs: Sustainable Development Goals
