# Supplementary material for: Supporting ALL victims of violence, abuse, neglect or exploitation: guidance for health providers
Source: BMC Int Health Hum Rights. 2018 Oct 19;18:39. doi: 10.1186/s12914-018-0178-y (PMC6194679; doi:10.1186/s12914-018-0178-y)
Supplement: Supplementary file 1 — Examples of differences between smaller groups of victims of violence, abuse, neglect or exploitation (VANE) in terms of identification, support and referral: an overview for health providers and social workers. (DOCX 36 kb) [file 12914_2018_178_MOESM1_ESM.docx]

# Additional file 1. Examples of differences between smaller groups of victims of violence, abuse, neglect and exploitation (VANE) in terms of *identification, support and referral*: an overview for health providers and social workers

**Table A1.1.** **Examples of differences between smaller groups of victims of violence, abuse, neglect and exploitation (VANE) in terms of *identification, support and referral*: an overview for health providers and social workers**

| *Group* | *More details about the group* | *Examples of signs that the person may be a victim of VANE* | *Examples of risk factors/groups* | *Examples of specific identification, support and referral considerations* |
| --- | --- | --- | --- | --- |
| Male victims of VANE | Violence against men remains a taboo in most societies, but many studies show it is very prevalent. All forms of violence, abuse and exploitation in this table also happen to men. | Signs for men largely resemble those for women, except that the violence more often goes unnoticed for a long time, because men often delay seeking help because of taboos and shame around being a victim of violence, and because health providers often do not consider the possibility of men being a victim of violence and hence infrequently ask men if they have been victimized | Risk factors and groups are largely the same for men and women: see the rows below for specific risk groups and factors per type of violence | - Very important to stress that violence against men is more prevalent than many people think and that it happens to men from all social strata and from all cultures, both to the victim and other involved professionals - Speak about feelings of shame and guilt - With sexually exploited men there may be a triple taboo: on homosexuality, on doing sex work and on being a victim |
| Elder abuse | Elder abuse concerns any type of violence, abuse or exploitation against people older than 65 years. Often, this concerns abuse by carers. | See other types of violence, in particular “Abuse by carers” | See other types of violence, in particular “Abuse by carers” | See other types of violence, in particular “Abuse by carers” |
| Abuse by carers | Abuse by carers may involve victims of any age and there are 6 types: neglect, psychological abuse, financial exploitation, rights violations, physical abuse, sexual abuse (often there is a combination)  Abuse by carers is subdivided into two distinct forms:  1. abuse, violence or exploitation  2. harm caused by informal, unpaid caregivers who are overworked and overstressed, without malevolent intent  This distinction is important because appropriate responses differ by form. | *Form 1: abuse, violence or exploitation:*  Physical injuries, especially when they cannot be logically explained or are diagnosed repeatedly; shows emotion without wanting to say why; person leaves anxious / depressive impression; sudden change in emotional welfare; extreme fear of certain people; fear of physical care; person and/or the household look neglected (e.g. empty fridge); money or property goes missing; increasing debts; the person is never allowed to speak alone to a health provider  *Form 2: harm caused by informal, unpaid caregivers without malevolent intent*  Most often psychological or physical abuse, neglect (e.g. empty fridge) or rights violations (e.g. bank cards are held by someone else leading to limited financial freedom). Signs among informal caregivers: the caregiver shows signs of being overworked; verbal abuse when health provider is present; caregiver appears not to care about the wellbeing of the patient; being late in calling in medical help; barring health providers from seeing the person. | Risk groups are people in care, e.g. elderly or people with disabilities. Additional risk factors:   - Increasing dependence - Disease that includes personality or behavioural changes, e.g. dementia - Family history of violence - Social isolation - Severe events (e.g. moving, death of a beloved, loss of job or divorce) | - Victims of abuse by carers may be reluctant in reporting violence out of loyalty to the (informal) caregiver or out of fear that the care might stop or the abuse might worsen after reporting it. - There may also be a decreased capacity to mention or report violence, to ask for help and to make decisions about next steps to follow (e.g. with people with dementia or mental disabilities) |
| Parent abuse | Parent abuse concerns violence that is committed by young people against their parents. Also known as: adolescent to parent violence, child-to-parent abuse, child-to-parent violence or battered parent syndrome. | Often feelings of shame and loyalty towards the child with parents, as well as a history of finding it difficult to set boundaries. As a result victims often come forward when the violence has existed for a long time. The violence may have exacerbated to extreme levels by then (arson, gun use, extortion, etc). First signs that something is wrong are often via a police report when the situation has become very bad.  Signs among perpetrators: sudden increase in verbal and physical violence from 14/15 years; bad school results and behaviour; no day activities; gaming and computer addiction; drug use; depression. | There are two main risk groups:  *Group 1*: divorced, often Caucasian mothers of young men who still live at home. Also happens with high SES. Often emerging psychiatric problems among perpetrator around 14/15 years.  *Group 2*: multi-problem families; history of trauma, domestic violence or child abuse; among perpetrator history of other types of violence, problems like debts or drugs, and behavioural problems at school | - There are few specific aid programmes available for this group, particularly for early detection and assistance. Involved professionals (GPs, youth care, safeguarding boards, teachers, police) should try to identify problems earlier and confer early on about possible interventions - The group where perpetrators are 18+ is particularly problematic because there are very few measures available and the perpetrators tend to refuse help |
| Human trafficking: across borders | Human trafficking is the exploitation of another person by means of the threat or use of force or other forms of coercion, of abduction, of fraud, of deception, of the abuse of power or of a position of vulnerability. There are different types of exploitation: sexual exploitation, forced labour (including also domestic work, forced begging or forced criminal activities such as drug smuggling), slavery or the removal of organs. Both men and women fall victim to trafficking.  This row focuses on cross-border human trafficking, see the rows below for more details on trafficking *within* countries. | Signs of human trafficking differ tremendously by person and by type of exploitation. General signs:  There is exploitation of a person by means of threat or use of force or other forms of coercion, of abduction, of fraud, of deception, of the abuse of power or of a position of vulnerability; person receives very low wages / another person benefits; passport is held by someone else; little freedom of movement, e.g. person is always accompanied / always picked up and brought to places; person did not arrange travel to new country him-/herself; dubious debts with employer; blackmail (with pictures or video, e.g. of a sexual nature); physical violence; threats (also against family); deception about nature of work or about relationship; person does not know work address; no fixed living space; fear of eviction from the country (for undocumented people); tattoos or voodoo-materials indicating dependency on someone; drug or alcohol addiction; complaints related to nature of work, e.g. anal complaints, repeated STDs, HIV, stomach aches for sexual exploitation. | - Dependent relationship / emotional dependence - Bad future prospects (e.g. no view to education or employment in origin country) - Low literacy - Family circumstances (e.g. orphans, broken homes) - Financial problems/ poverty - Social isolation/marginalization - Drugs addiction - History of violence or trauma (sexual abuse) - Homelessness - Mental health problems (esp. mild mental disability) | - Pay extra attention to the safety of yourself and the patient because of potentially involved criminal networks - Employ an external translator when needed, instead of using the person who accompanies the patient. Speak the patient alone if possible (or invent an excuse). Remember: traffickers may be family members! - These men and women are often not aware of their rights and of the trustworthiness of the police - They may not see themselves as victims of trafficking; development of a longer trusting relationship may be necessary to get someone to talk |
| Girls and boys below 18 years engaging in sex work (see also “Domestic human trafficking”) | This group overlaps with domestic human trafficking. However, minors also engage in sex work without being trafficked/groomed into it. Minors engaging in transactional sex may also be part of this group. This row focuses on the signs and referral considerations for boys and girls who are not victims of human trafficking but do engage in sex work (trafficking victims are discussed under “Human trafficking: domestic, within-country”). | Paid or transactional sex; rumours that the boy or girl is often seen with elderly men; needing money for drug or alcohol addiction; drug / alcohol use to be able to keep doing the work; debts; young age of starting sexual intercourse; large number of sexual partners; behavioural problems, depression, anxiety, suicide attempts; social isolation; relational and sexual problems / dysfunction; complaints related to nature of work, e.g. anal complaints, repeated STDs, HIV, stomach aches for sexual exploitation; parents with disapproving views of homosexuality (for boys); sudden change in possessions (luxury items) and available money | - Vulnerable position, e.g. handicapped, (undocumented) refugee, illiterate - Parents with little education - Living in large cities - Problematic family history - History of sexual violence - Homelessness or staying in youth care - For boys: being homosexual, bisexual or transgender | - These youths often do not see themselves as sexually abused and often do not see transactional sex or paid sex as sex work. As a result, they rarely come forward themselves with a demand for help specifically for sexual abuse (when they do they come for other things, see list of signs) - Quitting sex work can be difficult for youths because of a drop in income and because they have started to view men as clients |
| Human trafficking: domestic, within-country | This groups concerns boys and girls and men and women who are trafficked *within the country* (so in the case of the UK, this concerns UK residents). This is a large group in many countries (e.g. estimated at 56% in the Netherlands). Mostly this concerns sexual exploitation or criminal exploitation (e.g. using someone for smuggling drugs or to open bank accounts or telephone subscriptions) by either groups or individuals. “Grooming” is a method used by traffickers. | There is exploitation of a person by means of threat or use of force or other forms of coercion, of abduction, of fraud, of deception, of the abuse of power or of a position of vulnerability; increasing isolation from family and friends, being secretive about social contacts; leaving home or care; dependency on new relationship; having sex with friends or acquiring debts or phone subscriptions at boyfriend’s request; blackmail (with pictures or video, e.g. of a sexual nature); threats (also against family); vague explanations for physical injuries; sudden change in clothes, behaviour and possessions; having more than 1 phone; little freedom of movement: e.g. person is always accompanied / always picked up and brought to places; many new friends that do not fit old life; many fights at home; deteriorating school results and behaviour; psychosomatic complaints; complaints related to nature of work, e.g. anal complaints, repeated STDs, HIV, stomach aches for sexual exploitation; behavioural problems, depression, anxiety, suicide attempts; tattoos indicating dependency on someone; drug / alcohol use to be able to keep doing the work | - Multi-problem families - Broken homes - Low self-esteem - History of abuse/violence/trauma - Problematic teenagers - Little education - Age: 12-24 years - Mild mental disabilities - (undocumented) refugees - Honour based blackmail (see “Honour based violence”) - Homelessness - Social isolation - Having friends who are exploited | - Pay extra attention to the safety of yourself and the patient because of potentially involved criminal networks - Especially when grooming techniques are employed, this group may not see themselves as victims of exploitation. Development of a longer trusting relationship may be necessary to get someone to talk. Accept it when someone does not want to speak immediately, but keep offering a discussion about the topic. Be careful not to speak negatively about the groomer. |
| Sexual abuse or exploitation by gangs or groups | This group includes men and women who are sexually abused or exploited by groups or gangs. The group varies from youths being trafficked through grooming techniques into sex work to those being routinely raped by boys in their neighbourhoods.  This group is often conceptualized as containing only people below the age of 18 years, but people above 18 years of age also often fall victim to sexual abuse or trafficking for sexual exploitation (e.g. in the Netherlands this is the majority). | See “Domestic human trafficking” | - Living in a gang-affected neighbourhood - Having friends who are being sexually exploited - Dysfunctional / chaotic households - History of abuse or domestic violence - Low self-confidence or self-esteem - Low SES - Being in youth care - Having been trafficking - Being at risk of honour based violence - Homelessness | See “Domestic human trafficking” |
| Honour based violence | Honour based violence is psychological or physical violence to restore the honour of one’s family. This often concerns young girls and women, but boys/men may also fall victim to it.  There are different types of this violence: physical violence; psychological pressure (e.g. humiliation, threats); forced marriages; sending someone back to country of origin against their will; female genital mutilation; healing rituals; rejection from family; sexual abuse; and social control. Boys/men who refuse to enact honour based revenge may also fall victim. | Threat of breaking off or losing contact with family or being cast out; attempt to run away from home; always accompanied by family members at physician; not allowed to travel independently; request for evidence of virginity; not allowed to enter higher education; not allowed to own things that may hurt honour (e.g. Western music or movies); does not hold own passport; afraid to go on holiday (for fear of being left behind in origin country); a family discussion has been planned | Acute risk factors: sex before marriage; pregnant and unmarried; refusal of arrange marriage or planned return to origin country; homosexuality (or gossip about it); partner of other faith/race.  Other risk factors:  Family has a tradition of honour and/or is from a country with an honour based culture; history of violence in the family; family lacks integration into society; broader social environment also values honour | - When victims of honour based violence are identified by health providers, the violence has often already progressed extensively and it is crucial to act swiftly, e.g. in contacting the police or arranging alternative housing - Employ an external translator when needed, instead of using the person who accompanies the patient. Speak the patient alone if possible (or invent an excuse). Remember: this type of violence is typically perpetrated by family members |
| Forced marriages | Forced marriages are a form of honour based violence | See “Honour based violence” | See “Honour based violence” | See “Honour based violence” |
| Female genital mutilation (FGM) | FGM is a form of honour based violence | Planned visit abroad, in particular the family’s country of origin; staying away from health checks; staying away from school; long visits to the toilet; complaints about stomach aches and looking tired and pale; having been sick during holiday; UTIs, vaginal infections, STDs, problems with urination and menstrual problems; psychological consequences, e.g. anxiety for sexuality | - Family members who have undergone FGM - Family originates from country where FGM is practiced - Age: 4-12 years - Family and surroundings are positive about FGM - Family is not well integrated in new country - Note: FGM also happens in mixed marriages! | - See “Honour based violence” - People who have undergone FGM do not always link their physical or psychological complaints to their FGM; complaints are often seen as part of ‘being a woman’ - This group regularly lacks knowledge about the human body and its functions |

Legend: This table intends only to provide examples to give a general idea of these smaller groups of victims of violence; for more detailed advice around signs and referral considerations, we refer to the many documents there are about each specific group. The presence of one or even more signs and/or risk factors does not immediately indicate there is violence; it is the total of signs and risk factors that makes a health provider suspect there may be violence. Some groups overlap partially and signs and referral considerations may be shared by several groups. See Additional File 2 for the sources used to develop this table.

## References

1. *KNMG-Meldcode Kindermishandeling En Huiselijk Geweld*. Utrecht: KNMG; 2014.

2. *Model Reporting Code Domestic Violence and Child Abuse: Action Plan for Responding to Signs of Domestic Violence and Child Abuse*. Den Haag: Dutch Ministry of Health Welfare and Sport; 2013. http://www.government.nl/government/documents-and-publications/reports/2013/03/14/model-reporting-code-domestic-violence-and-child-abuse.html.

Sources used in developing Table A1.1:

- [www.signalenkaart.nl](http://www.signalenkaart.nl) (from the website: “In 2010 Kadera has developed the Indicator Checklist Domestic violence in cooperation with Dimence and de Kern. This list has been revised in 2013 and the Ministry of Health, Welfare and Sports has included it as a tool in the National toolkit working with the reporting code domestic violence and child abuse. In 2016 Kadera has reviewed the list again and developed it to the interactive Indicator Checklist Domestic violence and Child abuse with the help of FairWork, Pharos and Movisie.”)
- Signalerings-protocol mensenhandel. Veilig Thuis en Moviera. 2016. Ede.
- Marjan Wijers en Marcia Albrecht. Handreiking Signalering Mensenhandel voor werkers in de gezondheidszorg. SOA aids, 2014.
- Signaleringsprotocol Loverboys. Steununt Huiselijk Geweld. 2014.
- Minderjarige jongens die hun lichaam exploiteren: Jongensprostitutie. Ruilseks signaleren, bespreekbaar maken en motiveren tot stoppen. MOVISIE, 2013.
- Seksuele uitbuiting van jongens in Nederland. Paul van Gelder et al, SHOP Den Haag, Amsterdam, 2017.
- Hilde Bakker en Oka Storms. Factsheet De Meldcode bij (vermoedens van) eergerelateerd geweld. 2016. MOVISIE
- Handelingsprotocol VGV bij Minderjarigen: Uitleg en handvatten bij aanpak VGV voor Veilig Thuis, Raad voor de Kinderbescherming en Politie. PHAROS, 2016.
- PROTOCOL MELDCODE HUISELIJK GEWELD, IN HET BIJZONDER OUDERENMISHANDELING. ZorgvoorKennis, 2014.
- Pionieren in de mannenopvang. Anita C. Nanhoe. 2011. Gemeenten Amsterdam, Rotterdam, Den Haag, Utrecht.
- Bram Tuk en Stephanie Dauphin. De meldcode kindermishandeling en huiselijk geweld op scholen met asielzoekers en nieuwkomers: Stand van zaken en ervaringen met de aanpak van kindermishandeling. PHAROS, Utrecht, 2012.
- Oka Storms et al. Geweld is niet gewoon: vluchtelingen, asielzoekers en ongedocumenteerden: Ken uw recht – zoek hulp. Handboek. PHAROS en MOVISIE, Utrecht, 2014.
- Signalenkaart: voorkom ontspoorde mantelzorg. MOVISIE, Utrecht.
- Stappenplan Handelen bij ontspoorde mantelzorg. MOVISIE, Utrecht, 2015.
- Website: Wat is ontspoorde mantelzorg? 24 augustus 2016. MOVISIE, Utrecht. <https://www.movisie.nl/artikel/wat-ontspoorde-mantelzorg>
- Vink, R., Goes, A., Doornink, N. Broerse, A., Pannebakker, F., van der Zwan, R., & Schakenraad, W. (2014). Huiselijk geweld door kinderen en jongeren tegen hun ouders. Verkennend onderzoek. Utrecht/Leiden: Movisie/TNO.
- Huiselijk geweld door kinderen en jongeren tegen hun ouders: Kernbevindingen uit verkennend onderzoek. (2014). Utrecht/Leiden: Movisie/TNO.
- Herkenning en duiding van radicalisering. Nederlands Jeugd instituut. <https://www.nji.nl/nl/Download-NJi/Publicatie-NJi/Pol_Rad_Herkenning_duiding.pdf>
- Mensenhandel: vijfde rapportage van de Nationaal Rapporteur. Bureau Nationaal Rapporteur Mensenhandel, Den Haag.
- Alessandra Cancedda et al. Study on high-risk groups for trafficking in human beings: final report. European Commission, Migration and home affairs. 2015. doi:10.2837/59533
- Sue Berelowitz et al. “I thought I was the only one. The only one in the world” The Office of the Children’s Commissioner’s Inquiry into Child Sexual Exploitation In Gangs and Groups. Interim report. Office of the Childrens’Commissioner, November 2012.
- Website: Eergerelateerd geweld. Rijksoverheid. <https://www.rijksoverheid.nl/onderwerpen/eergerelateerd-geweld> geraadpleegd op 10 Nov 2017.
- Cecilia Menjivar and Olivia Salcido. Immigrant Women and Domestic Violence: Common Experiences in Different Countries. *Gender and Society*, Vol. 16, No. 6 (Dec., 2002), pp. 898-920. <https://www.peacepalacelibrary.nl/ebooks/files/Immigrant-Women-and-Domestic-Violence-Experiences-Menjivar.pdf>
- Samenwerkvel bij zorg over radicalisering. Radar, Bureau voor sociale vraagstukken. <https://www.radaradvies.nl/wp-content/uploads/sites/1/Samenwerkvel-Radicalisering-VOORBEELD.pdf>
- Magnus Ranstorp. Ran Issue paper: The Root Causes of Violent Extremism. Radicalisation awareness network Centre of Excellence, 2016. <https://ec.europa.eu/home-affairs/sites/homeaffairs/files/what-we-do/networks/radicalisation_awareness_network/ran-papers/docs/issue_paper_root-causes_jan2016_en.pdf>
- Anika Boersma et al. Signalenkaart mannelijke slachtoffers in de seksuele uitbuiting.
- Anniek Verhagen et al. Radicx: Vroegtijdige signalering van radicalisering. APS en KPC Groep. Utrecht, 2010. <http://www.advlimburg.nl/media/89722/radicx-tool.pdf>
- Slachtoffermonitor mensenhandel 2012-2016. Nationaal Rapporteur Mensenhandel en Seksueel Geweld tegen Kinderen, 2017, Den Haag. <https://www.nationaalrapporteur.nl/binaries/Slachtoffermonitor%20mensenhandel%202012-2016_Nationaal%20Rapporteur%20(i)_tcm23-285357.pdf>
- Website: “One in four 'extremists' reported to Government's deradicalisation programme are far-Right sympathisers, figures show”. Harry Yorke , 15 FEBRUARY 2017, The Telegraph. Accessed 28 november 2017. <http://www.telegraph.co.uk/news/2017/02/15/one-four-extremists-reported-governments-deradicalisation-programme/>
- Guidance on Prevent and the Channel Programme. Accessed 28 November 2017. <http://www.preventforfeandtraining.org.uk/sites/default/files/The%20Prevent%20Strategy%20and%20the%20Channel%20Programme%20in%20FE%20Colleges.pdf>

## Acknowledgements

We would like to thank for reviewing Table A1.1: Remy Vink, TNO; Annemiek Goes, MOVISIE; Tamara van Driel, CoMensha; Sandra van den Berg, CoMensha; Robert Weinberg, Oranje Huis Amsterdam/ Mannenopvang, Blijf Groep; Paul van Gelder, SHOP Den Haag; Evert Bloemen, PHAROS; Nico van Oosten, MOVISIE; and Anoushka Boet, MOVISIE.
