# Supplementary material for: Supporting ALL victims of violence, abuse, neglect or exploitation: guidance for health providers
Source: BMC Int Health Hum Rights. 2018 Oct 19;18:39. doi: 10.1186/s12914-018-0178-y (PMC6194679; doi:10.1186/s12914-018-0178-y)
Supplement: Supplementary file 2 — Sources used in the development of Table 1. (DOCX 47 kb) [file 12914_2018_178_MOESM2_ESM.docx]

# Additional file 2

Sources for Table 1 in main article, “One-year prevalence data of different types of violence, abuse, neglect or exploitation (VANE) worldwide and in the UK”

| **Intimate partner violence (IPV)** | |
| --- | --- |
| World (estimate) | “Latest comparable data for 87 countries from 2005 to 2016, including 30 from developed regions, shows that 19 per cent of girls and women aged 15 to 49 years have experienced physical and/or sexual violence by an intimate partner in the past 12 months”. The reported percentage was multiplied with 2016 world population estimate by the World Bank (7,442,135,580) to arrive at the number reported in Table 1 in the main article.  Source:  2017 High-Level Political Forum (HLPF) on Sustainable Development Thematic review of Sustainable Development Goal (SDG) 5: Achieve gender equality and empower all women and girls, a background note developed by members of the Executive Committee on Economic and Social Affairs (ECESA) Plus as a coordinated contribution by the UN system to the 2017 HLPF in depth review of SDG 5  <https://sustainabledevelopment.un.org/content/documents/14383SDG5format-revOD.pdf> |
| UK (estimate) | “This chapter presents findings from the year ending March 2016 Crime Survey for England and Wales (CSEW) self-completion module on intimate violence which is asked of adults aged 16 to 59. The module covers experience of emotional, financial and physical abuse by partners or family members, as well as sexual assaults and stalking by any person. This chapter also presents information on domestic abuse recorded by the police. The CSEW estimates that 7.7% of women and 4.4% of men experienced any type of domestic abuse in the last year. This is equivalent to an estimated 1.3 million female victims and 716,000 male victims.”  Source:  Compendium: Domestic abuse, sexual assault and stalking. UK Office for National Statistics. 9 February 2017.  <https://www.ons.gov.uk/peoplepopulationandcommunity/crimeandjustice/compendium/focusonviolentcrimeandsexualoffences/yearendingmarch2016/domesticabusesexualassaultandstalking> |
| UK (reported cases) | “For the 2015-16 reporting year there were 102,970 individuals with enquiries under Section 42 of the Care Act. Of these enquiries, 60 per cent were for females (61,985 enquiries) and 63 per cent of individuals at risk were aged 65 or over (65,085 enquiries).”  Source: Safeguarding Adults, Annual Report, England 2015-16, Experimental Statistics. NHS Digital, 2016. |
| **IPV against men** | |
| World (estimate) | data available for several countries, but no global data found.  From *The Oxford Handbook of Behavioral Emergencies and Crises*, edited by Phillip M. Kleespies, 2016, Chapter 14 “Intimiate Partner Violence: Evaluation of Victims and Perpetrators”, by T. Taft et al, page 198: “this report did not assess global prevalence of IPV against men, and to our knowledge no researchers or organizations have yet estimated global prevalence of IPV against men.” |
| UK (estimate) | See IPV general. |
| UK (reported cases) | “For the 2015-16 reporting year there were 102,970 individuals with enquiries under Section 42 of the Care Act. Of these enquiries, 60 per cent were for females (61,985 enquiries) and 63 per cent of individuals at risk were aged 65 or over (65,085 enquiries).”  Source: Safeguarding Adults, Annual Report, England 2015-16, Experimental Statistics. NHS Digital, 2016. |
| **Child abuse** | |
| World (estimate) | “Base case estimates showed a minimum of 50% or more of children in Asia, Africa, and Northern America experienced past-year violence, and that globally over half of all children—1 billion children, ages 2–17 years—experienced such violence.”  Source:  Global Prevalence of Past-year Violence Against Children: A Systematic Review and Minimum Estimates. Pediatrics. 2016;137(3):e20154079. <http://pediatrics.aappublications.org/content/early/2016/01/25/peds.2015-4079> |
| UK (estimate) | The 2013 NSPCC report “How safe are our children” reports the following estimated numbers for child maltreatment in 2011:   - 520,000 estimated victims of maltreatment by a parent or guardian - 260,000 estimated victims of maltreatment by an adult outside the home - 200,000 estimated children in need due to abuse and neglect   More recent reports by the NSPCC do exist but do not contain more recent estimates.  We enquired with the NSPCC about overlap between these numbers and if they could be added. They replied: “All the categories you enquired about will overlap. Category 3 “children in need due to abuse or neglect” relates to children identified as in need of support from children’s services because of abuse or neglect. You can read more about “children in need” in indicator 12 of How Safe are Our Children. Because of this, all the children in category 3 should be a subset of categories 1 and 2. There will also be some overlap between children who experienced abuse or neglect from a parent/guardian and those who experienced abuse or neglect from a non-resident adult. (…) At the NSPCC we use the estimate for abuse perpetrated by a parent/carer figure as our estimate for abuse experienced in a year, as the best estimate available without double counting, although this is likely to be an underestimate of the total extent of abuse. For example see here: <https://www.nspcc.org.uk/preventing-abuse/child-abuse-and-neglect/> “We estimate that over half a million children are abused in the UK a year.””    Therefore, the 520,000 number was reported in the table.  Source:  Lisa Harker, Sonja Jütte, Tom Murphy, Holly Bentley, Pam Miller and Kate Fitch. How safe are our children? National Society for the Prevention of Cruelty to Children (NSPCC), 2013. <https://www.nspcc.org.uk/globalassets/documents/research-reports/how-safe-children-2013-report.pdf> |
| UK (reported cases | “The number of children on child protection registers or subject to a child protection plan at 31 March 2016 (or 31 July 2016 in Scotland): 58,239”  Sources: <https://www.nspcc.org.uk/services-and-resources/research-and-resources/statistics/>; NSPCC, 2017. Child protection plan and register statistics: UK 2012 –2016. <https://www.nspcc.org.uk/globalassets/documents/statistics-and-information/child-protection-register-statistics-united-kingdom.pdf> |
| **Elder abuse** | |
| World (estimate) | “For this systematic review and meta-analysis, we searched 14 databases, including PubMed, PsycINFO, CINAHL, EMBASE, and MEDLINE, using a comprehensive search strategy to identify elder abuse prevalence studies in the community published from inception to June 26, 2015. Studies reporting estimates of past-year abuse prevalence in adults aged 60 years or older were included in the analyses.”  “The pooled prevalence rate for overall elder abuse was 15·7% (95% CI 12·8-19·3).”  Sources:   - World Health Organization <http://www.who.int/mediacentre/factsheets/fs357/en/> - Elder abuse prevalence in community settings: a systematic review and meta-analysis. Yon Y, Mikton CR, Gassoumis ZD, Wilber KH. Lancet Glob Health. 2017 Feb;5(2):e147-e156. <https://www.ncbi.nlm.nih.gov/pubmed/28104184>   The 15.7% reported above was multiplied with UN estimates for the number of people aged 60 years and older in 2015:  Persons aged 60 years or over (millions) in 2015: 900.9  Source: World Population Ageing report. United Nations, Department of Economic and Social Affairs, Population Division. 2015, New York. ST/ESA/SER.A/390. <http://www.un.org/en/development/desa/population/publications/pdf/ageing/WPA2015_Report.pdf> |
| UK (estimate) | “When the one year prevalence of mistreatment is broadened to include incidents involving  neighbours and acquaintances, the overall prevalence increases from 2.6% to 4.0%. This would  give a figure of approximately 342,400 older people subject to some form of mistreatment.”  Source:  Madeleine O’Keeffe et al. UK Study of Abuse and Neglect of Older People: Prevalence Survey Report. Comic Relief and the Department of Health. June 2007. <http://www.natcen.ac.uk/media/308684/p2512-uk-elder-abuse-final-for-circulation.pdf>  “As many as 370,000 older people have been abused in their own homes by a carer, relative or friend in the last year, according to figures, exposing what has been described as a "hidden national scandal".”  “The figures were compiled by the House of Commons library by extrapolating from a survey of 2,000 people who live in their own homes first carried out in 2007 by researchers at the National Centre for Social Research and King's College London. They found that the majority of abusers, 53%, were living in the respondent's house at the time of the abuse. Of those, 65% of perpetrators were recorded as having committed physical, psychological or sexual abuse.  The library analysis suggests that 371,900 people aged over 66 in the UK suffered abuse in their own home from a relative, carer or close friend in the last year, ranging from neglect and financial fraud to emotional abuse and physical or sexual assault. By 2020 the number is estimated to increase to 457,600; by 2030 the number is set to hit around 558,700. The projected increase in the number of people being abused correlates with the ageing of the country's population. People aged 85 and over are the fastest growing group in the population and are projected to increase substantially in numbers over the following decades.”  Source:  Hundreds of thousands of elderly people were abused last year. Daniel Boffey, Sunday 8 September 2013. The Guardian. <https://www.theguardian.com/society/2013/sep/08/elderly-abuse-carer-relative> Original report was not found and requested from the Parliament library, who could not provide the report. Therefore, the number was not included in the table. |
| UK (reported cases) | “For the 2015-16 reporting year there were 102,970 individuals with enquiries under Section 42 of the Care Act. Of these enquiries, 60 per cent were for females (61,985 enquiries) and 63 per cent of individuals at risk were aged 65 or over (65,085 enquiries).”  Source: Safeguarding Adults, Annual Report, England 2015-16, Experimental Statistics. NHS Digital, 2016. |
| **Abuse by carers** | |
| World (estimate) | No global data found |
| UK (estimate) | No UK data found |
| UK (reported cases) | Abuse by carers is variably defined: some refer to it only as abuse perpetrated by *unpaid* carers, others include also (or even focus specifically on) abuse by paid carers. Abuse by carers differs from elder abuse in that people of all ages may be victims. Depending on the definition that is used, it may also exclude abuse by paid or unpaid carers. Numbers for abuse by unpaid carers were not found. Numbers for abuse by paid carers were found and are reported in the Table:  “Between 2013-14 and 2015-16 there had been at least 23,428 safeguarding alerts across the UK, but only half the councils provided data.”  The reported number is for a two-year timespan, but only half of the UK councils are included in the reported number, so the number was left as is.  Source: website: Prosecutions 'rare' for abuse by home carers By Ben Robinson and Lesley Curwen. 28 February 2017. <http://www.bbc.com/news/uk-39042266> Based on a study conducted by BBC’s Radio 4 programme File on 4. |
| **Parent abuse** | |
| World (estimate) | Estimates for the prevalence of parent abuse vary greatly and thus were not reported.  Source:  Julie Selwyn and Sarah Meakings. Adolescent-to-Parent Violence in Adoptive Families. British Journal of Social work, 2016, 46(5), pages 1224-1240. <https://www.ncbi.nlm.nih.gov/pmc/articles/PMC4985723/> |
| UK (estimate) | No UK data found |
| UK (reported cases) | Parentline Plus investigated 29,972 calls to their helpline placed between October 2007 and June 2008 and found that: “Within the children’s behaviour category, 60% of these calls concerned verbal aggression which comprised 17% of all long calls. 30% concerned physical aggression, which comprised 8% of all long calls. The vast majority of this aggressive behaviour takes place at home – 86%, while 22% takes place at school and 1% in other locations. Boys and girls are as likely to exhibit such behaviour although there are differences when looking at physical and verbal aggression separately.”  In a later report, Parentline Plus reports: “A large number of calls to Parentline Plus’ free 24 hour a day telephone helpline have consistently concerned children’s behaviour: Between June 2008 and June 2010 (the period of time the statistics in this report cover, unless otherwise stated) 27% of the 83,469 long calls (classified as calls of a duration of 20 minutes or over) concerned children’s behaviour. Of these, 62% of callers were seeking advice about their child’s verbal aggression and 31% concerned physical aggression – 8% of all long calls to Parentline Plus.”  The number from the latest report was taken and divided by 2 to arrive at the number in the table.  Sources:   - YOU CAN’T SAY GO AND SIT ON THE NAUGHTY STEP BECAUSE THEY TURN ROUND AND SAY MAKE ME: Aggressive behaviour in children: parents’ experiences and needs. Parentlineplus. 2008 <https://helenbonnick.files.wordpress.com/2015/01/aggressive_behaviour_in_children.pdf> - WHEN FAMILY LIFE HURTS: Family experience of aggression in children. 2010. Parentline Plus. <https://www.familylives.org.uk/media_manager/public/209/Documents/Reports/When%20family%20life%20hurts%202010.pdf>   Other data sources:   - Another article mentions that in the greater London area alone, there were “1892 cases reported to the Metropolitan Police in 2009–2010, most of which involved violence against the person or criminal damage in the home. Our findings reveal that adolescent to parent violence is a gendered phenomenon: 87 per cent of suspects were male and 77 per cent of victims were female.” Source: Rachel Condry, Caroline Miles. Adolescent to parent violence: Framing and mapping a hidden problem. Criminology and Criminal Justice. Volume: 14 issue: 3, pages: 257-275. <https://doi.org/10.1177/1748895813500155> |
| **Immigrant or undocumented victims of violence** | |
| World (estimate) | No global data found |
| UK (estimate) | No UK data found |
| UK (reported cases) | “The Home Office report that on average 1,000 applications are made under the Domestic Violence Rule per annum. Of these approximately 35 – 50% are successful.”  This number only comprises the number of applications that are made for asylum based on the Domestic Violence Rule; the real number of immigrant and undocumented victims is likely to be much higher, particularly because this Rule only applies for people who have entered the country on a spousal visa (and of course the number also excludes all people who did are abused but to not apply for asylum under this rule). Therefore, this number is not reported in the table.  Source: Victims of Domestic Violence with No Recourse to Public Funds. Olvia Fellas and Harriet Wilkins. 2008. NRPF Network. <http://www.nrpfnetwork.org.uk/policy/Documents/nrpf_victims_dv_nrpf.pdf> |
| **Human trafficking / Forced Labour** | |
| World (estimate) | “24.9 million people were in forced labour. That is, they were being forced to work under threat or coercion as domestic workers, on construction sites, in clandestine factories, on farms and fishing boats, in other sectors, and in the sex industry. They were forced to work by private individuals and groups or by state authorities. In many cases, the products they made  and the services they provided ended up in seemingly legitimate commercial channels. Forced labourers produced some of the food we eat and the clothes we wear, and they have cleaned  the buildings in which many of us live or work.”  Source:  Global estimates of modern slavery: Forced labour and forced marriage*.* International Labour Organization (ILO), Geneva, 2017 ISBN: 978-92-2-130131-8 (print) 978-92-2-130132-5 (web pdf) 978-92-2-130135-6 (epub). <http://www.ilo.org/global/publications/books/WCMS_575479/lang--en/index.htm> |
| UK (estimate) | “The UK is a destination for men and women from Central and Eastern Europe, Asia, Africa and the Middle East often seeking better livelihood opportunities. In 2014, research was carried out in the context of the UK Government's Modern Slavery Strategy to estimate the scale of enslaved people living in the UK. The Home Office estimated as many as 10,000—13,000 potential victims of modern slavery in the UK, an estimate reflected in the 2016 Global Slavery Index.”  “The estimated confidence interval for the actual population size of potential victims of modern slavery in the UK (including the 2744 cases already known to the UK National Crime Agency (NCA)) is from 10 000 to 13 000, suggesting that the Strategic Assessment is aware of roughly 20–30% of all the potential victims in the United Kingdom in 2013. In round numbers, therefore, the dark figure is around 7000 to 10 000.”  Sources:   - The Global Slavery Index 2016 website, accessed 7 October 2017. <https://www.globalslaveryindex.org/country/united-kingdom/#footnote-[1>] - Kevin Bales et al. Modern slavery in the UK: How many victims? Significance, Volume 12, Issue 3, June 2015, Pages 16–21. <http://onlinelibrary.wiley.com/doi/10.1111/j.1740-9713.2015.00824.x/full>   The UK National Crime Agency (NCA) estimates that the number of victims of human trafficking in the UK is even larger:  ‘Will Kerr, the NCA’s director of vulnerabilities, said the figures were far higher than those identified by the system set up by the government to identify victims of trafficking, which stood at abut 3,800 in 2016. “It’s likely in the tens of thousands,” Kerr said. “The more we look for modern slavery the more we find evidence of the widespread abuse of the vulnerable. The growing body of evidence we are collecting points to the scale being far larger than anyone had previously thought.” '  This estimate was not admitted to the table because it was not a precise estimate and not as well documented as the other estimates available.  Source: Jamie Grierson. Tens of thousands of modern slavery victims in UK, NCA says. The Guardian, 10 August 2017. <https://www.theguardian.com/world/2017/aug/10/modern-slavery-uk-nca-human-trafficking-prostitution> |
| UK (reported cases) | “3,805 potential victims were submitted to the National Referral Mechanism in 2016”. This constitutes reported cases, so likely a strong underestimation.  Source: Modern Slavery and Human Trafficking: National Referral Mechanism Statistics - End of Year Summary 2016 Popular. 2017. UK National Crime Agency. 0380-MSHT. <http://www.nationalcrimeagency.gov.uk/publications/national-referral-mechanism-statistics/2016-nrm-statistics/788-national-referral-mechanism-statistics-end-of-year-summary-2016> |
| **Domestic human trafficking (within-country)** | |
| World (estimate) | The ILO Global estimates of modern slavery report reports that 23% of all victims of forced labour were living outside their country of residence during their exploitation (page 30).  Source:  Global estimates of modern slavery: Forced labour and forced marriage*.* International Labour Organization (ILO), Geneva, 2017 ISBN: 978-92-2-130131-8 (print) 978-92-2-130132-5 (web pdf) 978-92-2-130135-6 (epub). <http://www.ilo.org/global/publications/books/WCMS_575479/lang--en/index.htm> |
| UK (estimate) | No estimate was found for the UK. An estimate is available however for the Netherlands, where 56% of all victims of human trafficking are estimated to be victims of domestic trafficking. This percentage was multiplied with the estimate for total number of victims of human trafficking in the UK, described above, to arrive at the number in the table.  Source: Slachtoffermonitor mensenhandel 2012-2016. Nationaal Rapporteur Mensenhandel en Seksueel Geweld tegen Kinderen, 2017, Den Haag. <https://www.nationaalrapporteur.nl/binaries/Slachtoffermonitor%20mensenhandel%202012-2016_Nationaal%20Rapporteur%20(i)_tcm23-285357.pdf> |
| UK (reported cases) | A total of 326 victims with United Kingdom nationality was reported in the National Referral Mechanism report by the UK National Crime Agency in 2017. This number is an underestimation for domestic trafficking, because it excludes people who were trafficked domestically but are not UK nationals (e.g. people who entered the country under normal visas and were then trafficked within the UK). Moreover, this number seems small compared to the total number of registered victims in the UK (3,805), see above. In the Netherlands, 56% of all victims of human trafficking are victims of domestic trafficking. This makes one wonder if the UK National Referral Mechanism is failing to register a large group of victims of domestic trafficking.  Source: National Referral Mechanism Statistics. National Crime Agency, 2017. Reference: 0380-MSHT. <http://www.antislaverycommissioner.co.uk/media/1133/2016-nrm-end-of-year-summary.pdf>  Source: Slachtoffermonitor mensenhandel 2012-2016. Nationaal Rapporteur Mensenhandel en Seksueel Geweld tegen Kinderen, 2017, Den Haag. <https://www.nationaalrapporteur.nl/binaries/Slachtoffermonitor%20mensenhandel%202012-2016_Nationaal%20Rapporteur%20(i)_tcm23-285357.pdf> |
| **Girl and boys below 18 years engaging in sex work** | |
| World (estimate) | No global data found |
| UK (estimate) | No date for the UK found. Data were available for the Netherlands.  The estimate is that 1,500 boys below the age of 18 years engage in sex work annually in the Netherlands. This estimate was then adjusted using 2016 population numbers for the UK and the Netherlands to arrive at a number for boys under 18 years of age engaging in sex work: 5,785.  Sources:   - Minderjarige jongens die hun lichaam exploiteren: Jongensprostitutie. Ruilseks signaleren, bespreekbaar maken en motiveren tot stoppen, MOVISIE, 2013. <https://www.movisie.nl/publicaties/jongensprostitutie-minderjarige-jongens-die-hun-lichaam-exploiteren> - Vrijbuiters uitgebuit: Minderjarige jongens in de prostitutie. SWP, 2010.   Similarly, an estimate is available in the Netherlands for the number of girls. An older estimate from 1998 mentions 1,500 girls under 18 years of age that engage in sex work annually. A more recent estimate by the National Rapporteur mentions 1,363 victims of human trafficking for the purpose of sexual exploitation under the age of 18 years who were trafficked within the Netherlands, almost all of which were girls. This group does not overlap precisely with the group of underage sex workers (underage sex work does not always involve exploitation and thus is not always human trafficking), but it is commonly accepted in the Netherlands that underage sex work of girls consists mostly of human trafficking for sexual exploitation, while for boys there may be a broader spectrum of pay-dates, transactional sex and exploitation. Therefore, the estimate of 1,500 still seems valid, which was used and adjusted using 2016 population numbers for the UK and the Netherlands to arrive at a number for girls under 18 years of age engaging in sex work: 5,785.  Sources:   - Factsheet meisjesprostitutie: feiten en cijfers. MOVISIE, 2009. [https://www.movisie.nl/sites/default/files/alfresco_files/Factsheet%20meisjesprostitutie%20[MOV-226011-0.5].pdf](https://www.movisie.nl/sites/default/files/alfresco_files/Factsheet%20meisjesprostitutie%20%5bMOV-226011-0.5%5d.pdf) - Slachtoffermonitor mensenhandel 2012-2016. Nationaal Rapporteur Mensenhandel en Seksueel Geweld tegen Kinderen, 2017, Den Haag. <https://www.nationaalrapporteur.nl/binaries/Slachtoffermonitor%20mensenhandel%202012-2016_Nationaal%20Rapporteur%20(i)_tcm23-285357.pdf>   Total estimated number of girls and boys under 18 years of age who engage in sex work in the UK: 11,570. |
| UK (recorded cases) | No UK data found |
| **Sexual exploitation by gangs or groups / “grooming”** | |
| World (estimate) | No global data found |
| UK (estimate) | No UK data found |
| UK (reported cases) | “Based on evidence submitted to the CSEGG Inquiry, at least 16,500 children were identified as being at risk of child sexual exploitation during one year and 2,409 children were confirmed as victims of sexual exploitation in gangs and groups during the 14-month period from August 2010 to October 2011.”  “With regard to victims, agencies in 19 out of 39 police constabulary areas did not submit any  information on cases of child sexual exploitation in either gangs or groups in their responses to the call for evidence.”  The number reported in the table was arrived at by dividing 2,409 by 14 and multiplying with 12 to arrive at the reported number of cases in 1 year.  Source:  Sue Berelowitz et al. “I thought I was the only one. The only one in the world”. The Office of the Children’s Commissioner’s Inquiry into Child Sexual Exploitation In Gangs and Groups. Interim report. Office of the Children’s Commissioner, November 2012.  It is noteworthy that victims of grooming practices can be both boys and girls, and that they do not have to be below the age of 18 years. The report above focused on this group, but men and women older than 18 years are also regularly trafficked for sexual exploitation via grooming techniques. In the Netherlands, for example, estimates for the annual number of girls/women and boys/men who are trafficked *within the country* (so not across borders, also called domestic trafficking) for the purpose of sexual exploitation, which often happens via grooming techniques, comprised 1,542 adults and 1,363 girls and boys below the age of 18 years.  Source:  Slachtoffermonitor mensenhandel 2012-2016. Bureau Nationaal Rapporteur Mensenhandel en seksueel geweld tegen kinderen, Den Haag, 2017. |
| **Honour based violence** | |
| World (estimate) | data available for several countries, but no global data found |
| UK (estimate) | No UK data found |
| UK (reported cases) | “On Thursday 9 July 2015, the Iranian and Kurdish Women’s Rights Organisation (IKWRO), a leading women’s rights charity campaigning to end all forms of ‘honour’ based violence, including forced marriage, child marriage and female genital mutilation, publishes new research revealing that from 2010 to 2014 UK police have recorded more than 11,000 cases of ‘honour’ based violence. The figures, which were obtained under the Freedom of Information Act from 39 of 52 UK forces, show 11,744 incidents and crimes, which include abductions, beatings and even murders. The Metropolitan Police recorded the highest number at 2,188, followed by the West Midlands with 1,269 and Bedfordshire recording 1106. Over the five-year period ‘honour’ based violence cases were recorded in every single UK police force, demonstrating that this is a nation-wide problem.”  The number 11,744 was divided by the five year time span of the research to arrive at a one-year prevalence estimate.  Source: the Iranian and Kurdish Women’s Rights Organisation (IKWRO) website, accessed 7 October 2017. <http://ikwro.org.uk/2015/07/research-reveals-violence/> |
| **Forced marriages** | |
| World (estimate) | “15.4 million people were living in a forced marriage to which they had not consented. That is, they were enduring a situation that involved having lost their sexual autonomy and often involved providing labour under the guise of ‘marriage’.”  Source:  Global estimates of modern slavery: Forced labour and forced marriage*.* International Labour Organization (ILO), Geneva, 2017 ISBN: 978-92-2-130131-8 (print) 978-92-2-130132-5 (web pdf) 978-92-2-130135-6 (epub). <http://www.ilo.org/global/publications/books/WCMS_575479/lang--en/index.htm> |
| UK (estimate) | No UK data found |
| UK (reported cases) | “In 2016, the Forced Marriage Unit (FMU) gave advice or support related to a possible forced marriage in 1,428 cases. These figures include contact that has been made to the FMU through the public helpline or by email in relation to a new case.” So these are reported cases only.  Source:  Forced Marriage Unit Statistics 2016. Home Office and Foreign & Commonwealth Office. March 2017. <https://www.gov.uk/government/statistics/forced-marriage-unit-statistics-2016> |
| **Female genital mutilation** | |
| World (estimate) | “It is estimated that more than 200 million girls and women alive today have undergone female genital mutilation in the countries where the practice is concentrated. Furthermore, there are an estimated 3 million girls at risk of undergoing female genital mutilation every year. The majority of girls are cut before they turn 15 years old”.  Source:  World Health Organization website on Female Genital Mutilation (FGM), accessed 7 October 2017. <http://www.who.int/reproductivehealth/topics/fgm/prevalence/en/> |
| UK (estimate) | One year prevalence estimates or reported cases are not available for female genital mutilation in the UK, but they were available for the Netherlands. The estimate is that 40-50 girls are at risk of FGM each year in the Netherlands, especially when they visit their family’s country of origin. This estimate was then adjusted using 2016 population numbers for the UK and the Netherlands to arrive at the number in the table.  Sources:   - Handelingsprotocol Vrouwelijke Genitale Verminking bij minderjarigen, Pharos Centre of Expertise on Health for Migrants and Refugees, 2016. <http://www.pharos.nl/nl/kenniscentrum/algemeen/webshop/product/229/handelingsprotocol-vrouwelijke-genitale-verminking-bij-minderjarigen> - Female Genital Mutilation in the Netherlands Prevalence, incidence and determinants, by Marja Exterkate, 2013, Pharos Centre of Expertise on Health for Migrants and Refugees. <http://www.pharos.nl/documents/doc/webshop/vrouwelijkegenitaleverminkinginnederland-finalreportfgminnl1.pdf>   Other prevalence estimates are available for the UK:   - One report estimates that there are 24,000 girls at risk for type III FGM in the UK, but this at risk number is not reported in the table because questions have been raised about its validity, particularly about the fact that the report assumes that the likeliness of migrant populations performing FGM in the UK is the same as it is in the origin country.   Source: Website: The number of girls at risk of FGM is hard to estimate. Full fact, the Uk’s independent fact checking charity. <https://fullfact.org/health/number-girls-risk-fgm-hard-estimate/> . 2014.   - “an estimated 137,000 women and girls with FGM, born in countries where FGM is practised, were permanently resident in England and Wales in 2011”   Source: Alison Macfarlane, Efua Dorkenoo. Prevalence of Female Genital Mutilation in England and Wales: National and local estimates. City University London, London, 2015. <https://www.city.ac.uk/__data/assets/pdf_file/0004/282388/FGM-statistics-final-report-21-07-15-released-text.pdf> |
| UK (reported cases) | “In 18 newly recorded cases, the FGM was reported to have been undertaken in the United Kingdom, including 11 women and girls who were also reported to have been born in the UK.”  Source: Female Genital Mutilation (FGM) - April 2015 to March 2016, Experimental Statistics. NHS Digital. Publication date: 09:30 July 21, 2016.  Other reported case data were found:   - “The NSPCC launched its dedicated FGM helpline in June 2013 for anyone with concerns about FGM. People can contact the helpline by phone on 0800 028 3550 or by email. From 24 June 2013 to 31 January 2017 we received 1,564 contacts to the NSPCC Helpline on FGM. 35% of these contacts resulted in a referral to external agencies.”  Source: National Society for the Prevention of Cruelty to Children (NSPCC) website, accessed 05 October 2017. <https://www.nspcc.org.uk/preventing-abuse/child-abuse-and-neglect/female-genital-mutilation-fgm/fgm-facts-statistics/> - “Between April 2016 and March 2017 there were 9,179 attendances reported at NHS trusts and GP practices where FGM was identified or a procedure for FGM was undertaken.”   Source: Female Genital Mutilation (FGM) Annual Report 2016/17. NHS Digital, July 04, 2017. <http://digital.nhs.uk/catalogue/PUB30015> |
